# Tau and Membranes: Interactions That Promote Folding and Condensation

**DOI:** 10.3389/fcell.2021.725241

**Published:** 2021-09-21

**Authors:** Chad A. Sallaberry, Barbie J. Voss, Jaroslaw Majewski, Jacek Biernat, Eckhard Mandelkow, Eva Y. Chi, Crystal M. Vander Zanden

**Affiliations:** ^1^Department of Chemistry & Biochemistry, University of Colorado Colorado Springs, Colorado Springs, CO, United States; ^2^Division of Molecular and Cellular Biosciences, National Science Foundation, Alexandria, VA, United States; ^3^Department of Chemical & Biological Engineering, Center for Biomedical Engineering, The University of New Mexico, Albuquerque, NM, United States; ^4^Theoretical Biology and Biophysics Division, Los Alamos National Laboratory, Los Alamos, NM, United States; ^5^German Center for Neurodegenerative Diseases (DZNE), Bonn, Germany; ^6^Center of Advanced European Studies and Research (CAESAR) Center, Bonn, Germany; ^7^Department of Neurodegenerative Disease and Geriatric Psychiatry, Medical School, University of Bonn, Bonn, Germany

**Keywords:** tau, membrane, condensation, polyanionic surface, fibrils, phase separation, intrinsically disordered protein

## Abstract

Tau misfolding and assembly is linked to a number of neurodegenerative diseases collectively described as tauopathies, including Alzheimer’s disease (AD) and Parkinson’s disease. Anionic cellular membranes, such as the cytosolic leaflet of the plasma membrane, are sites that concentrate and neutralize tau, primarily due to electrostatic interactions with tau’s microtubule binding repeat domain (RD). In addition to electrostatic interactions with lipids, tau also has interactions with membrane proteins, which are important for tau’s cellular functions. Tau also interacts with lipid tails to facilitate direct translocation across the membrane and can form stable protein-lipid complexes involved in cell-to-cell transport. Concentrated tau monomers at the membrane surface can form reversible condensates, change secondary structures, and induce oligomers, which may eventually undergo irreversible crosslinking and fibril formation. These β-sheet rich tau structures are capable of disrupting membrane organization and are toxic in cell-based assays. Given the evidence for relevant membrane-based tau assembly, we review the emerging hypothesis that polyanionic membranes may serve as a site for phase-separated tau condensation. Membrane-mediated phase separation may have important implications for regulating tau folding/misfolding, and may be a powerful mechanism to spatially direct tau for native membrane-mediated functions.

## Introduction

The protein tau (Weingarten et al., 1975) forms aggregates observed in a variety of neurodegenerative diseases collectively described as tauopathies, including Alzheimer’s Disease (AD), argyrophilic grain disease, primary age-related tauopathy, frontotemporal dementia and parkinsonism linked to chromosome 17, frontotemporal lobar degeneration, Pick’s disease, corticobasal degeneration, progressive supranuclear palsy, Lytico-bodig disease (Parkinson-dementia complex of Guam), chronic traumatic encephalopathy, prion disease, Niemann-Pick disease type C1, San Filippo syndrome type B, Down’s syndrome (Lee et al., 2001; Verheijen et al., 2018). Furthermore, there is emerging evidence that Parkinson’s disease and Huntington’s disease may also be classified as tauopathies (Gratuze et al., 2016; Zhang et al., 2018). Tau aggregates were originally observed as β-sheet rich paired helical filaments (PHFs) and neurofibrillary tangles (Kidd, 1963), but a broader understanding of tau aggregation now includes oligomers (Flach et al., 2012; Tian et al., 2013). Tau-based pathogenic hallmarks spread around the brain correlating with neurodegenerative disease progression (Braak and Braak, 1991; Kaufman et al., 2018), and β-sheet rich misfolded tau has been proposed to seed fibrillation of intrinsically disordered tau monomers *in vitro* and *in vivo* (Clavaguera et al., 2009; Peeraer et al., 2015). However, it is unclear whether tau pathology spreads in a prion-like fashion proliferating neurodegeneration in human disease (Mudher et al., 2017). Certain missense mutations promote tau aggregation, and are likewise linked to disease pathogenesis (Hong et al., 1998; Barghorn et al., 2000). Tau also has splicing mutations that change the relative proportions of tau isoforms in the cell, and the full impact of these mutations on tau aggregation is not yet understood (Wang and Mandelkow, 2016).

One of tau’s major functions is the interaction with microtubules, consistent with its predominant localization in axons of mature neurons where it plays a major role in axonal transport (Weingarten et al., 1975; Witman et al., 1976; Hirokawa et al., 1996; Barbier et al., 2019). To achieve this role, tau also interacts with the subcortical actin meshwork underneath the plasma membrane to assist in anchoring the cytoskeleton to the plasma membrane (Baas et al., 1991; Biernat et al., 2002; Whiteman et al., 2009). The native functions of tau have yet to be fully defined, however, tau may also be involved in functions other than microtubule interactions and transport, such as neurite formation (Caceres and Kosik, 1990; Knops et al., 1991), proper formation of dendritic spines (Chabrier et al., 2014), regulation of synaptic vesicles (Largo-Barrientos et al., 2021), interactions with RNA (Wheeler et al., 2019; Lin et al., 2021), and others.

**FIGURE 1:**
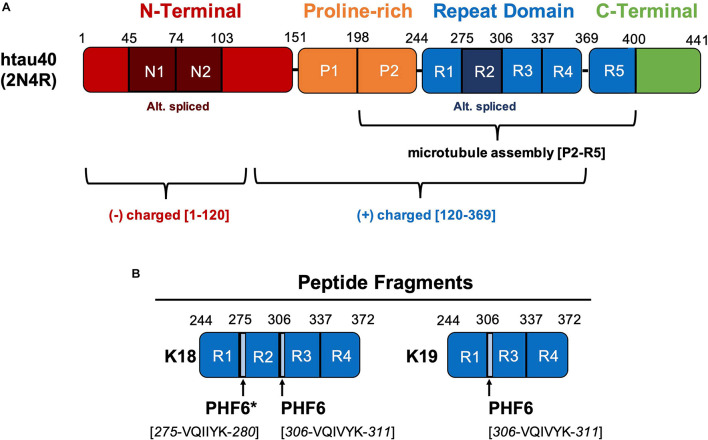
**(A)** Tau consists of 4 domains – the N-terminal, proline-rich, repeat domain (RD), and C-terminal domain. Residues 1–120 of the N-terminal domain bear an overall negative charge while residues 120–369, including the RD, are overall positively charged. Sub-domains N1, N2, and R2 are alternatively spliced; R2 corresponds to exon 10 of the gene. The longest central nervous system isoform, htau40, consists of 441 amino acids and is also notated as 2N4R indicating the presence of both N1 and N2 as well as R1, R2, R3, and R4. The C-terminal part (repeat domain + flanking domains P2 and R5) can be considered as MT-assembly domain as it binds strongly to MT and promotes their polymerization. The N-terminal half (N + P1) does not bind to MT (aka projection domain). The repeat domain RD forms the backbone of Alzheimer filaments. **(B)** Tau-based peptide fragments commonly used as models for assembly of β-sheet rich structures. The K18 and K19 fragments contain domains R1-R2-R3-R4 and R1-R3-R4 respectively. The hexapeptides PHF6 and PHF6* promote the assembly of β-sheets.

The major tau expressed in the human central nervous system (UniProtKB P-10636-8) encodes up to 441 amino acids, where the longest form of tau in the human central nervous system is commonly referred to as htau40 or 2N4R (Figure 1). Tau is alternatively spliced into six different isoforms representing the inclusion or omission of the N1, N2, and R2 domains, where N1 and N2 are in the N-terminal acidic projection domain (residues 1–151) and R2 is one of four pseudo-repeats in the repeat domain (RD) (Figure 1) (Mandelkow and Mandelkow, 2012; Guo et al., 2017). Sequence numbering of tau is usually quoted in terms of the 441 residue isoform, even when internal domains are not present. Larger isoforms of tau, such as “high molecular weight-tau” or “Big tau,” contain up to 758 residues and are expressed in the peripheral nervous system (P-10636-1) (Goedert et al., 1992). The initial 120 residues have an acidic character; amino acids 151–243 are termed a proline-rich domain characterized by seven Pro-XX-Pro (PXXP) motifs, which facilitate association with SH3 protein domains and microtubule assembly (Morris et al., 2011). The positively charged RD encompasses residues 244–368, containing four imperfectly repeated motifs R1–R4 of ∼31 residues. Microtubule binding is commonly ascribed to the RD, but it should be noted that strong binding requires components of the flanking PXXP and C-terminal domains (Butner and Kirschner, 1991; Gustke et al., 1994; Preuss et al., 1997). Although tau is an intrinsically disordered protein, several domains (R2, R3, R4, and parts of the R5) are known to aggregate into ordered β-sheet conformations (Crowther et al., 1989; Gustke et al., 1994; Yao et al., 2003; Sugino et al., 2009; Fitzpatrick et al., 2017). To study the role of the RDs in PHF aggregation, two model tau fragments are frequently used, containing R1–R2–R3–R4 or R1–R3–R4, termed K18 and K19, respectively (Gustke et al., 1994). Two key hexapeptides motifs are PHF6 (306-VQIVYK-311 at the start of R3) and PHF6^∗^ (275-VQIINK-280 at the start of R2) because they possess an enhanced propensity for β-structure and thus promote the assembly of β-sheets (von Bergen et al., 2000). Finally, the C-terminal domain, containing residues 369–441, is known to deter tau assembly into β-sheet rich structures (Abraha et al., 2000; Esposito et al., 2000; Mukrasch et al., 2009). R5 is considered a less-conserved 5th repeat motif, although parts of it are visible in the PHF structures obtained by cryo-EM. Tau is heavily post-translationally modified, with 45–51 experimentally observed phosphorylation sites, depending on the detection method and expression system (Drepper et al., 2020; Hanger, 2020; Wesseling et al., 2020). These phosphorylation sites act, in part, to regulate tau’s affinity to microtubules and/or tau’s self-assembly. Specific phosphorylation and acetylation (Min et al., 2015) sites are uniquely associated with disease pathology, although tau maintains a high level of phosphorylation even in healthy cells (Grundke-Iqbal et al., 1986; Wang et al., 2015). For this reason, recombinant tau containing some amount of phosphorylation mimics (Ser/Thr mutated to Glu) is likely a better model for cellular tau compared to a completely unphosphorylated protein (Guo et al., 2017; Wegmann et al., 2021).

The presence of tau aggregates is an important indicator of pathology in AD and other tauopathies. The initial trigger is unclear and might be variable, but tau assembly can be modeled by a process of tau monomers accumulating at a high local concentration, leading to reversible oligomerization or condensation, and eventually irreversible crosslinking and fibril formation. Polyanionic molecules and surfaces are known to promote tau condensation; early examples include heparin (Goedert et al., 1996) and arachidonic acid micelles or anionic beads (Wilson and Binder, 1997; King et al., 1999; Chirita et al., 2003) that are used to nucleate tau fibrils in solution. RNA is a biologically relevant polyanionic surface known to attract tau and generate condensates *in vitro* and *in vivo* (Kampers et al., 1996; Zhang et al., 2017; Lin et al., 2019). Microtubules have also been shown to nucleate tau condensates *in vitro* (Siahaan et al., 2019; Tan et al., 2019), however, the short dwell time for tau binding microtubules *in vivo* (Janning et al., 2014) may preclude a similar condensate assembly inside the cell. Preliminary evidence suggests that membranes may also promote tau condensation on a polyanionic surface.

Interest in tau-membrane interactions began in 1995 as part of general efforts to understand tau’s function in cells (Brandt et al., 1995). This work led to observations of tau interacting with proteins near membrane in cell models, namely neurons. Next came the discovery that arachidonic acid appeared to nucleate the assembly of tau into filaments, similar to the PHFs of AD (Wilson and Binder, 1997). This work developed into a thorough description of how anionic surfaces of vesicles could nucleate PHF assembly, including the role of each tau domain and the impact of lipid composition (Elbaum-Garfinkle et al., 2010). Tau assembly on membranes led to investigation of membrane-mediated toxicity, exploring concepts of tau-induced membrane leakiness to small molecules (Künze et al., 2012) and perturbation to membrane structure (Jones et al., 2012). Another avenue of interest was to determine the cell biology of tau transfer across cell membranes, which was hypothesized to be the reason for spreading of AD pathology in the brain (Brunello et al., 2020).

The purpose of this review is to examine the role of biological membranes as polyanionic surfaces (Platre and Jaillais, 2017) that have the potential to attract tau, catalyze folding, and promote condensation. In this way, membranes have the potential to nucleate pathological folding of assembled tau aggregates, or to concentrate tau at the cell periphery to facilitate its native membrane-mediated functions. We will consider tau’s native interactions with membrane proteins and the significant body of *in vitro* work documenting the structural changes tau undergoes when interacting with anionic lipids. While most research documents tau’s interactions with anionic charged phospholipid headgroups, we will also examine the evidence for tau’s direct interactions with hydrophobic lipid tails. Finally, we will discuss the emerging evidence for direct observations of tau condensation on membrane surfaces.

## Main Body

### Tau Has Native Interactions With Lipids and Membrane Proteins

**FIGURE 2:**
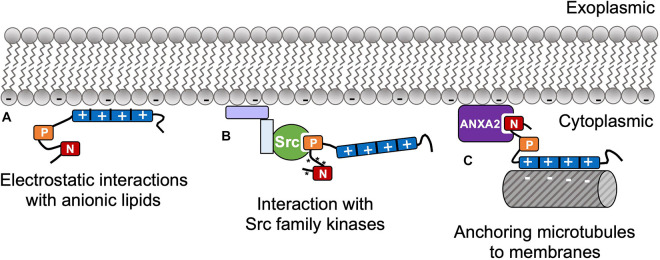
**(A)** Tau has direct native interactions with membranes including electrostatic affinity of basic side chains around the repeat domain with anionic lipids through the RD. Tau also has indirect membrane interactions via membrane-associated proteins, e.g., **(B)** PXXP motif interactions with SH3 domains in Src family kinases, which regulate tau’s N-terminal phosphorylation (* indicates phosphorylation); and **(C)** anchoring microtubules to the plasma membrane via N-terminal interactions with Annexin A2 (ANXA2).

The first indication of tau-membrane interactions was in 1995, which was attributed to tau interactions with cortical actin and associated proteins, rather that direct interactions with lipids (Brandt et al., 1995). From Wilson and Binder (1997) came the first observation that purified arachidonic acid could nucleate rapid growth of tau fibrils (King et al., 1999). It is highly inefficient to grow fibrils from pure isolated tau in solution due to the protein’s high charge and solubility that preclude the homogeneous nucleation that precedes fibril growth. A few years later, Chirita et al. (2003) documented that tau fibrillation could be induced by anionic micelles and vesicles, and the link between lipids and tau pathology began to emerge. In addition to pathological interactions, tau also has native interactions with membranes that are important for healthy cellular function. Several groups have documented evidence that was interpreted to be tau serving as a linker protein to anchor microtubules to cellular membranes; this function is implicated in neuritic development (Brandt et al., 1995) and in microtubule interactions with Golgi membranes (Farah et al., 2006). Taken together, these studies show tau has clear affinity for the plasma membrane, both through direct binding to lipids and through interactions with membrane-bound proteins (Figure 2).

Tau’s affinity for lipids is highly dependent on electrostatic interactions and phospholipid headgroup composition. In vesicle binding studies, K19 strongly prefers anionic lipids over zwitterionic lipids, even in the presence of 100 mM NaCl. K19 binds to 20% anionic 1-palmitoyl-2-oleoyl-sn-glycero-3-phosphoserine (POPS) vesicles with an affinity of 0.93 μM, while the affinity was reduced 60-fold to 55.3 μM for binding to pure zwitterionic 1-palmitoyl-2-oleoyl-glycero-3-phosphocholine (POPC) vesicles. A 10-fold reduction in NaCl concentration doubled tau’s affinity to the anionic lipid mixture (to 0.42 μM), further supporting that tau participates in electrostatic interactions with the lipid headgroups (Künze et al., 2012). These observations were corroborated with fluorescence correlation spectroscopy studies that showed K18 also exhibits strongest binding to anionic vesicles (Elbaum-Garfinkle et al., 2010). Binding was further enhanced in an acidic environment (Elbaum-Garfinkle et al., 2010), likely because K18’s overall charge increases from +10.3 to +16.5 as the pH is reduced from 7.4 to 5.0. To determine which microtubule binding RDs were important for membrane affinity, each domain (R1, R2, R3, and R4) was isolated and tested using isothermal titration calorimetry. Only R2 and R3 produced considerable enthalpic binding to 80:20 DOPC:DOPG (1,2-dioleoyl-sn-glycero-3-phosphocholine:1,2-dioleoyl-sn-glycero-3-phospho-[1′-rac-glycerol]) vesicles, and no domains showed any interaction with DOPC vesicles (Dicke et al., 2017). Overall, electrostatic interactions in the R2 and R3 domains are a primary determinant in tau’s binding affinity to membranes, and membrane interactions are highly dependent on charge modulators such as membrane composition, pH, and ionic strength. It should be noted that K18 and K19 are useful tau fragments for aggregation studies, but they may not necessarily represent the behavior of whole tau. Future *in vitro* studies should also consider the recent evidence that tau is natively phosphorylated in the cell, and a pseudophosphorylated tau would be more representative than a completely unphosphorylated tau.

In addition to the RD driving tau’s membrane affinity, there is significant evidence that the N-terminal projection domain also interacts with the membrane. This was first documented in a 1995 study with neuronal cells overexpressing tau’s N-terminal domain along with endogenous tau. These cells developed neurites abnormally, suggesting an interference with tau’s native role in microtubule interactions at the cell periphery. The authors interpreted that over-expressed N-terminal domain competitively bound the neural plasma membrane and inhibited the proper function of endogenous tau, although other explanations are also possible (Brandt et al., 1995). Analysis of isolated tau fractions from neuroblastoma cells (Arrasate et al., 2000) and rat cortical neurons (Pooler et al., 2012) reveals that membrane-bound tau is reactive with the tau-1 antibody, which specifically binds tau dephosphorylated in the 192–204 region [complementary to the phospho-epitope of AT8 (Szendrei et al., 1993)]. Furthermore, membrane-bound tau did not bind to antibodies AT8 and PHF-1, which specifically bind phosphorylated tau at Ser202/Thr205 and Ser396/404, respectively (Pooler et al., 2012). This antibody-binding profile suggests lack of phosphorylation at some sites, but it does not preclude phosphorylation at other sites. Furthermore, due to the variable affinity and limited sequence coverage of antibodies, a complementary approach such as mass spectrometry would improve the accuracy of tau phosphorylation site determination. In another study with full-length tau (441 amino acids), phosphorylation was found to dynamically modulate htau40’s association with membranes (Ekinci and Shea, 2000). Treatment with okadaic acid, which inhibits phosphatases, reduced the membrane-bound fraction from 22% of total cellular tau down to 7% (Arrasate et al., 2000). Inhibition of casein kinase 1 and glycogen synthase kinase-3 promoted additional tau binding to the membrane in a reversible manner (Pooler et al., 2012). This effect was also recapitulated with phosphorylation mimics, where 5, 6, or 18 Ser/Thr residues were mutated to Glu in both the N-terminal and C-terminal domains of tau. Interestingly, there was no difference in the C-terminal mutations, but the N-terminal phosphorylation mimics significantly reduced tau’s association with the membrane (Pooler et al., 2012). This indicates that kinases and N-terminal phosphorylation play a significant role in regulating tau’s membrane association by control of electrostatic interactions with anionic membranes.

To regulate phosphorylation, tau interacts with a plethora of membrane-affiliated kinases that are involved in cell signaling cascades. Tau has affinity to SH3 and SH2 domains (Usardi et al., 2011), which are adaptor domains that guide many cell-signaling kinases to their appropriate binding target (Koch et al., 1991; Schlessinger, 1994). The SH3 domain recognizes PXXP motifs, which are abundant in the proline-rich region of tau, especially Pro216 within the PXXP(213–216) motif (Usardi et al., 2011). It is well documented that tau specifically binds SH3 domains of some proteins, but not all, in the Src kinase family, including cSrc, Fgr, phospholipase Cγ1, phosphatidylinositol-3-kinase, Grb2, and fyn (Lee et al., 1998; Reynolds et al., 2008).

Fyn kinase is one member of the Src kinase family that has received much attention for its interactions with tau. Fyn has many diverse functions and binding partners, but a few key roles include cytoskeletal remodeling and neurite development. Fyn associates with membranes via myristoyl and palmitoyl anchors, and the majority of the protein is clustered in microdomains in cytosolic leaflet of the plasma membrane (Resh, 1998). In primary cortical neurons, fyn phosphorylates tau and drives tau to also localize in membrane microdomains (Usardi et al., 2011). In addition to tau’s native interactions with fyn, many studies have focused on blocking tau-fyn interactions to inhibit toxic impacts from amyloid beta (Aβ). Early 2002 reports showed that one facet of Aβ toxicity is neurite deformation, and that this can only occur in the presence of tau (Rapoport et al., 2002). Aβ oligomers activate fyn, which in turn causes binding and pathological phosphorylation of tau near the plasma membrane (Rush et al., 2020). It was further shown that Aβ toxicity could be ameliorated using a peptide inhibitor that binds the 5th and 6th PXXP motifs in the tau protein, therefore blocking Fyn’s binding (Rush et al., 2020). Overall, this suggests that fyn is responsible for Aβ-mediated pathological phosphorylation of tau, and that inhibiting this interaction may be a viable therapeutic target.

Tau has native functions with other membrane-bound proteins beyond kinases, specifically low-density lipoprotein receptor-related protein 1 (LRP1), muscarinic receptors, and annexin A2 (ANXA2). Tau is primarily considered as an intracellular protein, but it also is found in nanomolar concentrations extracellularly (Yamada et al., 2011). Although tau does not have an extracellular signaling sequence, it is suspected to traverse the cell membrane via exocytosis. The function of extracellular tau is still under investigation (Yamada, 2017), but there is evidence that tau has specific interactions with extracellular membrane proteins. Tau interactions with LRP1 were found to regulate endocytic uptake of tau. In neuroglioma cells and stem-cell derived neurons, LRP1 knockdown reduced tau uptake, and LRP1 downregulation caused reduced tau spread between neurons mouse model (Rauch et al., 2020). Muscarinic acetylcholine receptors are G-protein coupled receptors involved in a plethora of membrane-mediated signaling events and biological processes, and M1 and M3 are highly abundant types of muscarinic receptors (Felder, 1995). M1 and M3 activation cause intracellular calcium release (Lanzafame et al., 2003), and they are hypothesized to be involved in extracellular tau’s ability to increase intracellular calcium (Gómez-Ramos et al., 2006). Antagonists of M1 and M3 receptors were able to block this toxic function of tau (Gómez-Ramos et al., 2008). Thus, it is expected that extracellular tau has direct interactions with M1 and M3 to cause a neurotoxic rise in intracellular calcium levels.

In addition to muscarinic receptors, tau also has direct interactions with ANXA2, which is an adaptor protein that links the cytoskeleton to the membrane (Gerke et al., 2005). These interactions are likely important for localizing tau in axons and linking axon microtubules to the cell membrane. ANXA2 interacts with tau via eight conserved residues on tau’s far N-terminus (Gauthier-Kemper et al., 2018), and importantly, this interaction leaves the RD unaffected, allowing for binding of both ANXA2 and microtubules simultaneously. In a contrasting study, the R406W (Froelich et al., 1998) mutation in tau’s RD reduced membrane interactions, and the authors suggested this was due to reduced affinity for ANXA2 (Gauthier-Kemper et al., 2011). This interaction needs further exploration, but it is clear that tau interactions with ANXA2 promote its localization to the plasma membrane. Overall, tau has membrane-mediated interactions that serve as mechanisms for intracellular signaling and are important for native neuronal functions.

### Tau Folds Upon Binding the Membrane

There is substantial evidence that tau changes conformation when it binds to phospholipid membranes. The resulting structures range from α-helical (Georgieva et al., 2014; Snow et al., 2019) to β-sheet rich (Elbaum-Garfinkle et al., 2010; Fanni et al., 2019; Majewski et al., 2020), and they are more compact than the aqueous state of tau (Jones et al., 2012; Künze et al., 2012). Initially, these partially-folded tau structures rest on the surface of the membrane (Georgieva et al., 2014), but they appear to embed further into the phospholipid membranes over time (Künze et al., 2012). The emerging hypothesis is that membranes may play a role in tau neuropathology via two mechanisms – nucleating tau fibrils at the membrane (Elbaum-Garfinkle et al., 2010; Fanni et al., 2019; Majewski et al., 2020), and membrane permeabilization caused by tau interactions (Flach et al., 2012; Patel et al., 2015). In this section, we provide a review of studies that aim to determine the structural changes tau undergoes upon binding the membrane. Historically, many studies were performed with tau fragments K18 or K19 in an effort to partition the 441 amino acid protein into amyloidogenic and non-amyloidogenic fragments. However, future studies must also consider the contributions from the N-terminal domain, as this region has been shown to be important for membrane interactions and tau assembly. Furthermore, while *in vitro* studies provide a foundation for studying tau interactions with membranes, they cannot fully recapitulate the complex environment found in the cell. The native cellular environment contains hundreds of unique lipid types, a membrane surface decorated with proteins, and many other cellular proteins (tubulin, actin, and others) that may compete with tau-membrane interactions. Future research in this area should work toward examining tau-membrane interactions in the native cellular environment.

**FIGURE 3:**
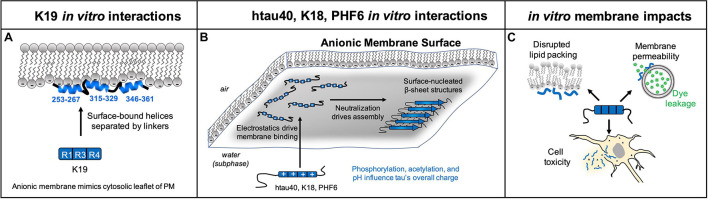
*In vitro* experiments have documented that membranes promote tau folding. **(A)** K19 forms a partially helical structure when interacting with a pure anionic membrane, where experiments with model anionic membranes were designed to mimic the cytoplasmic leaflet of the plasma membrane. **(B)** htau40, K18, and PHF6 assemble into β-sheet rich structures when binding a membrane containing >25% anionic lipids. Tau’s membrane affinity is driven by favorable electrostatic interactions with anionic lipid headgroups, and assembly on the membrane is promoted by charge neutralization. **(C)** *In vitro* experiments show tau can have deleterious impacts on membrane structure of synthetic vesicles including disrupted lipid packing, membrane permeability to small molecule reporters, and cell toxicity resulting from interactions with tau fibrils and aggregation intermediates. Note that *in vivo* tau is on the inside of vesicles involved in transfer of tau in or out of cells.

Biophysical characterization of tau K19, which contains domains R1, R3, and R4, showed that the peptide forms an α-helical structure during interactions with anionic phospholipid headgroups (Figure 3A). Circular dichroism of K19 revealed a distinct shift from an aqueous disordered state to a partially-folded state upon binding anionic vesicles, with ∼27% α-helical and ∼25% β-sheet content in the membrane-bound form (Barré and Eliezer, 2006; Künze et al., 2012). During K19 interactions with anionic sodium dodecyl sulfate micelles, the hexapeptide PHF6^∗^ formed an α-helix, while the hexapeptide PHF6 formed β-strands (Eliezer and Barr, 2013). Despite this β-sheet content, no fibrils grew from incubation of 6 μM K19 and 600 μM 4:1 POPS:POPS LUVs for 7 days (Künze et al., 2012). Binding to anionic micelles and vesicles revealed that K19 formed three distinct helices encompassing residues 253–267, 315–329, and 346–361, which are the same residues proposed to mediate tau binding to microtubules (Barré and Eliezer, 2006). These amphipathic helices rest on the surface of the membrane, and they are connected by flexible linkers that allow for different inter-helix distances and variable compaction of membrane-bound K19 (Georgieva et al., 2014). This interaction caused perturbation of lipid structure by altering the headgroup organization, and also by increasing lipid chain disorder, causing an area per molecule increase of 6.5 Å^2^ per lipid (Künze et al., 2012).

In contrast to K19, tau K18 (containing domains R1–R2–R3–R4) readily formed Thioflavin T-sensitive fibrils upon binding to anionic vesicles (Figure 3B) (Elbaum-Garfinkle et al., 2010). Fluorescence correlation spectroscopy with labeled K18 or lipids revealed a two-step process of (1) K18 binding to vesicles, followed by (2) membrane-nucleated K18 aggregation (Elbaum-Garfinkle et al., 2010). Fibrillation seemed to be largely driven by electrostatic screening and the propensity for phospholipids to concentrate the peptide at the membrane surface. At pH 5, aggregation was observed from 270 nM K18 at a 1:98 protein to lipid ratio, whereas pH 7.4 required micromolar K18 concentrations and a ratio of 1:36. At an acidic pH, the membrane is capable of concentrating the protein 5 orders of magnitude above the bulk solution (Elbaum-Garfinkle et al., 2010). These results indicate that it is highly likely that surface-induced concentration of K18 plays a role in promoting the peptide’s aggregation.

The hexapeptide PHF6 (VQIVYK) rapidly forms fibrils upon binding to anionic membranes, showing that this motif is sufficient for fibrillation independent of the other residues comprising K18 (Fanni et al., 2019). PHF6 with N-terminal acetylation showed rapid insertion into an anionic 1,2-dimyristoyl-sn-glycero-3-phospho-[1′-rac-glycerol]) (DMPG) monolayer at the air/water interface and exhibited lipid-nucleated fibrillation within 1 h, while fibrillation in the absence of membranes required several hours and was less robust (Fanni et al., 2019). Liquid surface X-ray diffraction revealed that acetylated PHF6 formed a repeating unit with 4.7 Å lattice spacing, which coincides with the spacing between β-strands in β-sheet rich fibrils (Fanni et al., 2019). Protein structures protruded 24 Å from the surface of the membrane (Fanni et al., 2019), which agrees with a PHF6 fibril model predicted from atomic-resolution structures of htau40 neurofibrillary tangles (Fitzpatrick et al., 2017). Studies were also performed with non-acetylated PHF6, which bears an additional positive charge on the N-terminus compared to the acetylated form. Non-acetylated PHF6 exhibited stronger binding to membranes, but it was unable to assemble into β-sheet rich structures (Fanni et al., 2019). Furthermore, non-acetylated PHF6 was less toxic than acetylated PHF6 in cell-based assays. Overall, PHF6 rapidly forms fibrils on an anionic membrane surface, and fibrillation caused toxicity in neuroblastoma cells. It should be noted that PHF6 is a useful model for studying membrane binding and assembly, but it does not recapitulate the complexity of whole tau.

htau40 can also form membrane-nucleated fibrils, first shown in 2003 by electron microscopy images of fibrils growing from the surface of phosphatidylserine vesicles (Chirita et al., 2003). htau40 readily interacts with anionic DMPG monolayers, but interacts significantly less with cationic or zwitterionic membranes (Jones et al., 2012). β-sheet-rich structures were observed after 12 h of incubation with an anionic monolayer, evidenced by grazing incidence X-ray diffraction studies (Majewski et al., 2020). This interaction reduced the amount of liquid condensed domains observed in the membrane, thus causing a major disruption in membrane organization (Jones et al., 2012; Majewski et al., 2020). Liquid surface X-ray reflectivity showed that htau40 predominantly embeds into the lipid head groups, but also slightly penetrates into the tails over extended incubation times (Jones et al., 2012). Even in the absence of lipids, tau is highly surface active and readily adsorbs to an air/water interface (which can serve as a model of a perfect hydrophobic interface), forming a dense 16 Å protein layer (Jones et al., 2012) that is highly compacted compared to the aqueous state of tau (Bernadó et al., 2007). This provides a partial explanation for tau interaction with lipid tails despite it being highly charged and soluble.

To isolate the contribution from individual domains of htau40, additional studies were performed with K18 and an htau40 phosphorylation mimic with seven Ser/Thr to Glu mutations in the domains flanking the repeats, characteristic of tau derived from AD brain (Majewski et al., 2020). K18 exhibited increased membrane interactions, and it formed β-sheet-rich aggregates faster and in higher amounts than htau40. The phosphorylation mimic had less binding to an anionic membrane, but interestingly, it exhibited enhanced aggregation compared to htau40 (Majewski et al., 2020). Although tau phosphorylation reduces binding to an anionic membrane, it enhances fibrillation by reducing repulsion between positively charged protein monomers. Overall, membrane-mediated fibrillation appears to be driven by two independent processes - membrane binding, and misfolding and assembly into fibrils. This idea of two separate processes is supported by several independent studies with htau40 (Majewski et al., 2020), K18 (Elbaum-Garfinkle et al., 2010; Majewski et al., 2020), and PHF6 (Fanni et al., 2019).

It is clear that tau membrane binding and fibrillation can disrupt membrane organization, but there is also evidence that non-fibrillar tau structures permeabilize membranes. A canon of amyloidogenic diseases (including tauopathies) is that protein oligomers, rather than fibrils, are suspected to be the most toxic. In a study comparing the toxicity of tau monomers, aggregation intermediates, and fibrils, the aggregation intermediates were the most toxic to SH-SY5Y neuroblastoma cells and caused the most leakage from phosphatidic acid vesicles (Flach et al., 2012). Testing each microtubule binding domain in isolation revealed that R2 and R3 had the strongest ability to cause calcein leakage when interacting with anionic membranes (Dicke et al., 2017), while none of the domains caused any leakage with zwitterionic membranes (Dicke et al., 2017). Thus, anionic lipids seem to be required for tau’s non-fibrillar membrane-mediated toxicity.

Non-fibrillar tau species are hypothesized to form pores in membranes, thus providing one possible explanation for their membrane disruption activity and toxicity. Tau monomers were observed to form ion channel pores in 1,2-dimyristoyl-sn-glycero-3-phosphoserine (DMPS) bilayers, as detected with atomic force microscopy imaging and planar lipid bilayer electrical recording experiments (Patel et al., 2015). In another study, interaction of helical tau K19 with DMPS vesicles resulted in 95% vesicle leakage within 2 min, while in contrast, K19 interactions with 4:1 1,2-dimyristoyl-sn-glycero-3-phosphocholine (DMPC)/DMPS vesicle mixtures required 12 min to cause 15% leakage, indicating high anionic lipid content is required for tau to cause membrane leakiness (Künze et al., 2012). The idea of tau-induced pore-formation and membrane leakiness is still highly debated, and more work is needed to clarify if this phenomenon can be observed *in vivo*.

Overall there are a few key conclusions that can be made from *in vitro* biophysical characterization of tau interactions with membranes. The first is that tau prefers to bind anionic membranes, especially under acidic conditions. Favorable electrostatic interactions with phospholipid headgroups drive tau to the membrane, and factors such as histidine charge and phosphorylation are important for regulating tau’s membrane affinity. Once tau binds the membrane, it is highly prone to misfold and self-assemble into β-sheet-rich fibrillar species. The factors influencing fibrillation are separate from those driving membrane binding, for example, charge neutralization decreases tau’s affinity for anionic membranes, but it enhances oligomer and fibril assembly while tau is concentrated at the membrane surface. The hexapeptide PHF6 is capable of nucleating membrane-mediated fibrillation, and fibrillation has also been observed for K18 and htau40. These fibrillar tau species disrupt native membrane packing and they are toxic to cells (Figure 3). In addition, there is significant evidence for membrane-mediated toxicity caused by non-fibrillar aggregation intermediates.

In addition to the significant body of *in vitro* work characterizing tau interactions with membranes, a few *in vivo* studies also address tau structure on the membrane and how this interaction influences membrane integrity. Electron microscopy images of neurons from patients with advanced AD showed tau PHFs originated from cytomembranes, suggesting that membranes may serve as nucleation sites for β-sheet rich tau structures (Gray et al., 1987). Furthermore, PHFs isolated from AD brains were found to contain small amounts of cholesterol, phosphatidylcholine, and sphingolipids (Goux et al., 1995, 2001; Gellermann et al., 2006). In co-cultures of neurons and astrocytes, membrane-active tau oligomers were found to cause neuronal cell death. Extracellular tau aggregates were able to incorporate into the plasma membrane, disrupt native ion currents, and alter membrane potential, suggesting the downstream toxicity was mediated through deleterious tau-membrane interactions (Esteras et al., 2021). In mitochondria isolated from SH-SY5Y human neuroblastoma cells, oligomeric tau exhibited cardiolipin-mediated interactions with the mitochondrial membrane. These interactions resulted in organelle swelling and loss of membrane potential, indicating deleterious interactions with the mitochondrial membrane (Camilleri et al., 2020). Although these mitochondrial experiments were performed *in vitro*, they are much closer to modeling the cellular environment due to isolation of the mitochondria directly from neuroblastoma cells. Overall, more *in vivo* work is needed to investigate tau interactions with membranes in the native cellular environment.

### Tau Has Direct Interactions With Anionic Lipids That Lead to Secretion

**FIGURE 4:**
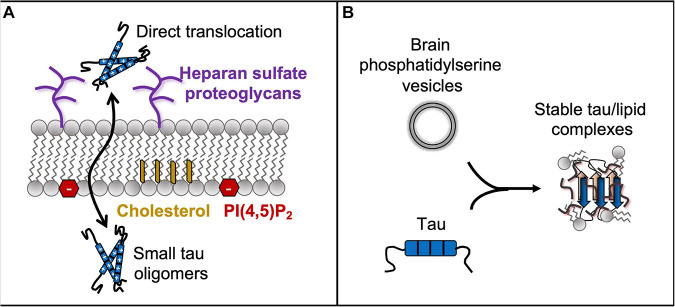
Tau has favorable interactions with entire lipid molecules, including the phospholipid heads and tails, which is inferred by tau’s direct translocation across the membrane **(A)** and by formation of stable tau-lipid complexes **(B)**.

Tau pathology spreads throughout the brain during neurodegenerative disease progression following the classically described Braak staging (Braak and Braak, 1991). This spreading has been described using a template-assisted model, where misfolded tau from one cell is able to pass to neighboring cells and spread throughout the brain as a hallmark in AD (Clavaguera et al., 2009; Gerson and Kayed, 2013; Brunello et al., 2020). This hypothesis of pathological tau spreading is still a matter of debate, but it is clear that the spread of tau pathology can serve as a useful diagnostic marker for AD disease progression. Tau is found in extracellular space both as a free protein and contained within vesicles. Even in non-pathological conditions, monomeric tau is secreted into the extracellular space. Canonical transport mechanisms may facilitate this process, with exocytosis likely responsible for the free tau observed in extracellular space at nanomolar concentration (Yamada et al., 2011). Tau does not have a signaling sequence to direct its trafficking outside of the cell, but despite this fact, exocytosis of tau has been observed. Some extracellular tau is found inside extracellular vesicles that bud from the plasma membrane (Dujardin et al., 2014; DeLeo and Ikezu, 2018). Finally, tau has been observed to spread via tunneling nanotubes formed between cells. These well-documented transport mechanisms of tau have been recently reviewed and verified in many cell lines and animal models for tau pathology (Yamada, 2017; Vasili et al., 2019; Brunello et al., 2020). In addition to these traditional modes of secretion, evidence is emerging that tau may have direct interactions with lipids that support translocation across the plasma membrane (Figure 4) (Katsinelos et al., 2018; Merezhko et al., 2018). Furthermore, favorable interactions between tau and whole lipid molecules, including both the phospholipid headgroup and the tail, are interesting from a structural biology perspective.

One non-canonical method of tau secretion is through direct passage across the cell membrane. This concept of direct translocation across the membrane is not entirely new as it is an accepted transport mechanism for fibroblast growth factor 2 (Zehe et al., 2006). In 2018, tau’s direct passage across the membrane was documented by two independent research groups. Initial observations showed that conventional secretion inhibitors (brefeldin A and monensin) failed to decrease in extracellular tau SH-SY5Y cells (Katsinelos et al., 2018). Furthermore, tau secretion did not respond to modulating ATP levels, suggesting that secretion was independent of any ATP-mediated process such as vesicle fusion. In fact, chemical induction of exocytosis by ionomycin decreased tau excretion (Merezhko et al., 2018). These findings support a hypothesis for a non-canonical secretion mechanism for tau.

Further exploration suggests this mechanism depends on tau interactions with phosphatidylinositol 4,5-bisphosphate [PI(4,5)P_2_] lipids and extracellular sulfated proteoglycans. Results from *in vitro* vesicle assays showed that tau only bound PC vesicles when they were mixed with PI(4,5)P_2_ lipids, and that this interaction also caused membrane disruption as evidenced by dye leakage assays. Furthermore, blocking PI(4,5)P_2_ headgroups with the antibiotic neomycin caused a concentration-dependent reduction in secreted tau levels in SH-SY5Y cells (Katsinelos et al., 2018). Tau secretion is also mediated by availability of sulfated proteoglycans on the extracellular leaflet of the plasma membrane. Tau is significantly associated with the extracellular side of the plasma membrane, suggesting it is anchored there before release into the cell culture medium. Treatment with sodium chlorate was used to inhibit production of sulfated glycosaminoglycans by 40%, which in turn caused a 30% reduction in the level of extracellular tau in SH-SY5Y cells and mouse primary hippocampal neurons (Katsinelos et al., 2018). Tau phosphorylation was also linked to secretion, with hyperphosphorylation leading to higher levels of secreted extracellular tau. Finally, these secreted tau proteins are taken up by neighboring cells and can trigger pathogenic tau aggregation.

Although the previous study was performed with tau monomers, the authors specifically state that their results do not exclude the possibility of small oligomers being secreted in this way. In the 2018 study published by Merezhko et al. (2018), tau passage through the membrane was found to be dependent on tau oligomerization and membrane fluidity. In mouse N2A neuroblastoma cells with overexpressed tau, 99% of secreted tau oligomers were in a vesicle-free form and 80% of all secreted tau was found as dimers, trimers, or tetramers. Small puncta were observed near the surface of the cell, which co-stained with antibodies for phosphorylated tau (AT8 and PHF13) and tau oligomers (T22), but could not be stained with Thioflavin S. These puncta had a mean diameter of 23 nm, and 90% of all particles were smaller than 40 nm. Tau aggregation inhibitors reduced tau secretion 35–94% depending on the inhibitor used, with the strongest reduction in tau secretion caused by epigallocatechin gallate in rat primary cortical neurons with endogenous tau expression (Merezhko et al., 2018).

Cholesterol and sphingomyelin were also requirements for tau secretion, indicating that tau release is promoted by liquid-ordered phase domains in the membrane. Treatment with methyl-β-cyclodextrin to reduce cell membrane cholesterol decreased tau secretion by 47%, while increasing cellular cholesterol increased tau secretion by 75%. The impact of sphingomyelin was also tested by adding sphingomyelinase or myriocin, and both treatments reduced tau secretion by 36%. Docosahexaenoic acid was used to reduce membrane order, and it inhibited tau secretion by 94% (Merezhko et al., 2018). Finally, the authors also observed that tau secretion depends on glycosaminoglycans. Treatment with sodium chlorate caused a 37% decrease in tau secretion, and treatments with heparinase I or heparinase III, which is used to cleave heparin sulfate-type sulfated glycans, reduced tau secretion by 25% (Merezhko et al., 2018). Previous work had suggested that tau can also enter cells through interaction with exoplasmic membranes containing heparan sulfate (Holmes et al., 2013; Rauch et al., 2018), so it is possible this mechanism of direct translocation across the membrane is occurring in both directions. Overall, tau oligomers are favored for secretion via interaction with lipid rafts and extracellular heparin sulfate proteoglycans.

A persistent question that follows the discovery of direct translocation across the membrane is how tau is interacting with lipids to facilitate this process. Tau is highly charged and soluble, so it is not immediately obvious how it might transverse the hydrophobic tails of the plasma membrane. One possible answer to this question is the formation of stable tau-lipid complexes that spread pathologically from cell to cell (Ait-Bouziad et al., 2017). The existence of these complexes indicates that tau is able to form stable interactions with hydrophobic lipid tails, suggesting a thermodynamically favorable mechanism for tau to directly translocate across the membrane.

These stable tau/lipid complexes also have important implications for spreading tau neuropathology. Co-incubation of tau and anionic phosphatidylserine vesicles at a 1:20 molar ratio caused conversion of the vesicles into homogenous small round spheres with a size of ∼10 nm in a few hours. These structures can be formed at physiological tau concentrations (500 nM) and with brain lipid extract, although in lower quantities. The complexes equilibrated to a protein to lipid ratio of 5:150 htau40:lipid (complex molecular weight of 335 kDa) and 7:140 K18:lipid (230 kDa), and they co-stained with the MC1 antibody raised against tau PHF from AD brains (Ait-Bouziad et al., 2017). NMR results show that PHF6 forms the core of these protein/lipid complexes and is immobilized in β-strand-like secondary structures. PHF6^∗^ and nine residues following the hexapeptide are also immobilized in the oligomer core, although the secondary structure is less defined. K18 formed complexes with the same efficiency as full-length tau. However, the deletion of the R2 domain decreased complex formation. At pH 6.0–6.5, the tau/lipid complexes converted into filament-like aggregates, suggesting they are able to nucleate tau polymerization. Interestingly, mutation of V287E (in R2), V318E (in R3), and K311A in htau40 caused significant reduction in membrane binding, but did not inhibit fibril formation, effectively decoupling these two behaviors of tau. Finally, these complexes were taken up by neighboring neuronal cells after 1 day of incubation and they caused apoptotic cell death. The tau/lipid complexes co-stained with early endosomal markers, but not late endosomal markers, suggesting they are taken up by endocytosis and then escaped the endocytic pathway (Ait-Bouziad et al., 2017). Overall, these findings document transiently stable interactions between tau and whole lipid molecules, including hydrophobic lipid tails. This indicates that when considering tau-membrane associations, it is important to consider additional interactions beyond pure electrostatic affinity to anionic lipid heads.

### Membrane-Induced Tau Condensation

Biological condensates have gained a lot of attention in recent years, and tau has not escaped this renaissance in biological phase separation science. Condensation is the ability of a molecule to selectively concentrate in defined foci, allowing for spatial organization that is not established by membrane-bounded organelles, such as mitochondria or peroxisomes (Lyon et al., 2020). Condensation encompasses a spectrum of phases ranging from liquid to solid, with each phase defined by the amount of disorder and dynamic motion in the condensate. One example of a solid condensate is amorphous aggregates made of the protein TDP43 (Boeynaems et al., 2018). Liquid–liquid phase separation (LLPS) is characterized by free mobility of molecules inside a phase-separated droplet. Stronger intermolecular interactions promote formation of gels or solids along the condensate spectrum, and protein gels are often nucleated by a preliminary LLPS state (Harmon et al., 2017). There is precedent for proteins forming condensates on membranes (Banjade and Rosen, 2014; Case et al., 2019; Huang et al., 2019), and emerging evidence suggests that tau may also participate in membrane-induced condensation.

In brief, a few general principles describe a molecule’s propensity to condense. Crowding agents, such as polyethylene glycol, promote LLPS, and unsurprisingly, phase separation is dependent on protein concentration (McCarty et al., 2019). LLPS is often promoted by a protein primary sequence that balances electrostatic residues separated by linkers (Wang et al., 2018). As such, salts typically inhibit LLPS. Interestingly, zinc promotes tau LLPS, but this is not observed for other divalent transition metal ions (Singh et al., 2020). Tau condensation has been well-documented and is reviewed in full elsewhere (Ukmar-Godec et al., 2020; Zbinden et al., 2020; Zeng et al., 2020), necessitating only a brief recount here with an emphasis on the interplay between tau condensation, fibrillation, and the impact of polyanions.

Full-length htau40 phase separates at concentrations as low as 2 μM protein, which is the concentration of tau found in neurons (Gamblin et al., 2003), when combined with 6% polyethylene glycol and 10 mM NaCl (Boyko et al., 2019). Tau LLPS is highly regulated by biological controls such as alternative splicing, phosphorylation, and other factors regulating tau’s charge such as pH. Alternative splicing of the R2 domain decreases propensity for LLPS (Ambadipudi et al., 2017), and phosphorylation at positions S262, S324, and S356 promoted K18 LLPS at 2 μM, compared to the >10 μM required for LLPS in unphosphorylated K18 (Ambadipudi et al., 2017).

Condensation is particularly common among intrinsically disordered proteins (Harmon et al., 2017; McCarty et al., 2019), and one hypothesis, among many, posits that protein phase separation may serve an important role in regulating protein folding and misfolding (Lyon et al., 2020). It follows that LLPS may be a critical cellular control mechanism to inhibit the misfolding of tau that is a hallmark of disease. An alternative hypothesis is that tau condensation may drive the nucleation of aggregates due to the high localized protein concentration and charge neutralization. This hypothesis is supported by several accounts that tau forms β-sheet rich oligomers nucleated from phase separated droplets (Ambadipudi et al., 2017, 2019). Pathogenic tau mutations G272V, P301L, and ΔK280 do not change tau’s propensity for LLPS but they do promote fibrillation from the phase-separated state, which implies that there are distinct factors driving condensation compared to those that drive fibrillation (Boyko et al., 2020). Disease-relevant tau mutations are generally understood to promote interactions between tau molecules in the phase-separated liquid droplet, thus enhancing the propensity for oligomerization or fibrillation of tau once it is organized into phase-separated droplets (Boyko et al., 2020; Kanaan et al., 2020). In one study aiming to clarify the relationship between LLPS and fibril formation, tau droplets were induced by polyanions in many conditions, and each condition was screened for fibrils to determine if droplet phase-space coincided with fibril nucleation (Lin et al., 2020). While there were many conditions that promoted both LLPS and fibril formation, fibril growth was not specifically linked to LLPS (Lin et al., 2020). The exception was in tau LLPS formed in the presence of high-salt conditions, where electrostatics were not expected to be the dominant interaction driving tau association. Under these specific conditions, the authors did conclude there was direct correlation between LLPS and fibril formation (Lin et al., 2020).

Polyanions such as heparin induce LLPS, but tau also forms condensates around abundant polyanions found in the cell, including RNA (Zhang et al., 2017; Lin et al., 2019) and tubulin (Hernández-Vega et al., 2017; Tan et al., 2019; Savastano et al., 2020). These condensates readily form under physiological conditions using recombinant tau, and due to their salt-dependence, they are thought to be in-part dependent on electrostatic interactions. It should be noted that further research is required to understand the impact of phosphorylation on tau condensation, as most studies have been performed using recombinant tau that lacks the high degree of phosphorylation observed in the native cellular environment. Surface-bound macromolecule condensates are expected to form as an extension of multi-layer adsorption, and they may form at lower concentrations than what is needed for bulk-phase separation (Mitchison, 2020). Furthermore, surface-bound condensates are thermodynamically “pinned” to their site of origination, which suggests their potential to serve as a powerful tool for spatial organization within the cell (Mitchison, 2020). Membrane surfaces have been documented as sites that promote biomolecular phase separation in several cellular contexts. A few key examples of cellular processes that are influenced by membrane-induced phase separation include actin polymerization at the membrane surface, activation of T-cells, and synaptic vesicle organization (Snead and Gladfelter, 2019). It is natural to ask whether tau may also form condensates on the surface of anionic membranes, as membranes also serve as sites that neutralize and concentrate tau. Given the overlapping conditions for condensation and fibril growth in the presence of polyanions, LLPS at the membrane surface may initiate membrane-nucleated fibrillation. Furthermore, condensation may also regulate non-pathogenic tau by concentrating it at the membrane surface and promoting interactions with membrane-associated proteins such as ANXA2 and Src kinases.

**FIGURE 5:**
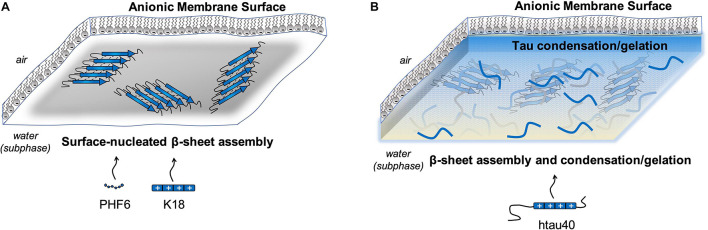
**(A)** PHF6 and K18 interact with anionic membranes to form β-sheet rich fibrillar structures. **(B)** Full-length htau40 condenses to form a macroscopic gel when interacting with anionic membranes, in addition to nucleating β-sheet assembly.

There is emerging experimental evidence to suggest that membranes may promote tau condensation and/or phase separation. In one report, htau40 interacted with anionic lipids to form a macroscopic gel after incubating the protein and lipid monolayer together overnight (Majewski et al., 2020). The gel was formed from 1 μM protein in a pure water subphase with no crowding agents, such as polyethylene glycol. Previous reports have described that tau rapidly transitions from a LLPS state to a gel-like state within 30 min, suggesting that over time the tau molecules develop additional interactions driving higher-ordered assembly (Wegmann et al., 2018). In this membrane-mediated gel state, protein β-sheet rich aggregates were detected by grazing incidence X-ray diffraction after 10 h of incubation with the membrane, suggesting fibrillation and gel formation are co-existing at the membrane surface (Majewski et al., 2020). This macroscopic gel was not formed from K18 or PHF6 interactions with anionic phospholipid monolayers (Fanni et al., 2019; Majewski et al., 2020), thus it is apparent that full-length tau is required for phase separation under these conditions. K18 and PHF6 both readily form fibrils on a DMPG membrane, indicating that while phase separation for full length htau40 is co-existing with fibrillation, phase separation does not need to precede fibrillation (Figure 5).

Membrane-mediate tau condensation has been observed at the nuclear envelope in brain tissue from AD patients (Kang et al., 2021). The droplets were originally observed from staining patient brain samples with FTLD-MAPT-P301L tauopathies, and the observation was then recapitulated in cell-based model systems with a P301L tau mutation. The droplets were able to rapidly recover from photobleaching and fuse with other droplets, confirming they were indeed liquid. Tau condensation also caused nuclear deformation and bubble-like protrusions in the nuclear envelope (Kang et al., 2020). These tau condensates served as sites for fibril nucleation when the droplet grew larger than 3 μm, as detected by Thioflavin S staining. This may provide an explanation for the classic neurofibrillary tangles observed around the cell nucleus in tauopathies. Finally, tau condensation also disrupted nuclear trafficking, which eventually led to cell death (Kang et al., 2020).

One other report suggests tau condensation onto membranes, but the protein’s physical state in this system is not immediately clear. In this study, atomic force microscopy was used to characterize htau40 deposition onto supported lipid bilayers assembled from brain total lipid extract (Mari et al., 2018). The authors observed non-fibrillar blotchy patches of amorphous tau deposited onto the membrane in an ion-dependent manner. Full-length tau promoted the best membrane coverage and growth of the amorphous protein patches compared to smaller patches formed by K18 (Mari et al., 2018). This membrane attachment was highly dependent on ion content, with sodium and lithium driving attachment and potassium and rubidium inhibiting it (Mari et al., 2018). This ion-dependence is reminiscent of tau’s preference for zinc, but not other transition metals, to promote LLPS in aqueous phase (Singh et al., 2020). Amorphous tau assembly onto brain lipid extracts may be indicative of tau condensation on the membrane, but more investigation is required.

Overall, it is a reasonable hypothesis that cellular membranes may serve as polyanionic surfaces that promote tau condensation and phase separation. Tau condensation on the cytoplasmic leaflet of the plasma membrane may be important for several reasons: (1) formation of tau aggregates in the cytoplasm; (2) formation of aggregates that may cross the cell membrane and be released into the extracellular space; (3) condensation may be a mechanism that deters tau misfolding; or (4) concentrated tau at the membrane surface may promote native tau-membrane interactions. Tau condenses on other cellular polyanions, including nucleic acids and microtubules, so it is logical to expect the same for cellular membranes that display clusters of regions of anionic charge. Anionic membranes are known to concentrate and neutralize tau, as shown by the body of work documenting tau assembly using *in vivo* and *in vitro* systems. The cytosolic leaflet of the plasma membrane is the most negatively charged membrane in the cell, driven by high amounts of anionic phosphatidylserine and phosphatidylinositol lipids (Platre and Jaillais, 2017). These anionic lipids in the membrane surface are often bound by cationic proteins, suggesting tau could also bind the membrane surface through electrostatic interactions. The experimental evidence to support membrane-mediated tau condensation is only emerging, but we anticipate this will become a significant consideration for native tau function and pathological aggregation in the future.

## Conclusion and Future Perspectives

Tau interacts with several membrane proteins to carry out its native function. Through interactions with ANXA2, tau is recruited to the cell periphery to anchor axonal microtubules to the plasma membrane (Gauthier-Kemper et al., 2018). Tau also has affinity to the ubiquitous SH3 domain found in many proteins, including Src kinases, which may serve as a target for ameliorating Aβ toxicity and tau hyperphosphorylation (Usardi et al., 2011). In addition to binding membrane proteins, tau also has direct interactions with anionic lipids through electrostatic interactions with phospholipid headgroups. The lysine/arginine-rich RD and proline-rich domains allow tau to bind membranes with as little as 20% anionic lipid content, which is similar to that found on the cytosolic leaflet of the plasma membrane (Künze et al., 2012). N-terminal phosphorylation at residues 50, 69, 111, 153, and 181 also regulate membrane affinity, which further highlights the importance of kinase interactions in tau’s ecosystem (Pooler et al., 2012).

*In vitro* experiments show that tau adopts a compacted and partially folded structure upon binding anionic membranes. Two clear structures have been consistently documented – a partially helical structure and a β-sheet rich structure that is capable of nucleating fibril growth from the membrane. The isolated RD K18 is both necessary and sufficient for tau’s membrane binding and fibrillation (Elbaum-Garfinkle et al., 2010). It is well documented that anionic membranes can nucleate tau fibrillation, however, there are independent factors driving membrane affinity compared to the factors driving tau-tau protein interactions. Strong cationic charge in the RD and flanking regions drives tau binding to the anionic membranes, but this same charge deters self-association events such as aggregation and fibrillation (Majewski et al., 2020). The R2 binding domain seems to promote β-sheet formation on the membrane surface, as studies with K19 (lacking the R2 domain) produced helical structures instead of β-sheet rich structures. These helices lie flat on the membrane surface and do not show much penetration into the lipid tails, indicating their membrane affinity is primarily driven by interactions with phospholipid head groups (Barré and Eliezer, 2006). This membrane-mediated helical structure is especially interesting because K19 is known to form β-sheet rich fibrils when mixed with heparin (Siddiqua and Margittai, 2010). With knowledge that the N-terminal domain also contributes to membrane binding, future research should prioritize experiments with whole tau protein, as the fragments K18 and K19 are incapable of recapitulating the behavior of the tau’s N-terminus. Furthermore, *in vitro* experiments cannot fully replicate the complex environment of the cell membrane, and future work should aim to explore tau structure and membrane interactions in a native cellular environment. In some *in vitro* vesicle studies, tau also causes membrane-mediated toxicity by causing membrane permeability to small reporter molecules (Flach et al., 2012; Künze et al., 2012; Patel et al., 2015). In comparison to the body of work on Aβ oligomers and their interactions with membranes, relatively little is known about how tau causes membrane-mediated toxicity. This will be an important avenue for future exploration.

In addition to surface interactions with lipid headgroups, tau can also stably associate with hydrophobic phospholipid tails. Two groups have documented direct translocation of tau monomers and oligomers across the membrane (Katsinelos et al., 2018; Merezhko et al., 2018). This translocation is mediated by anionic PI(4,5)P_2_ lipids on the cytoplasmic leaflet of the plasma membrane and by sulfated proteoglycans on the extracellular leaflet. Tau also forms stable protein-lipid complexes that are capable of completely disassembling vesicles and transforming them into an array of smaller particles (Ait-Bouziad et al., 2017). These complexes stably contain both lipids and proteins, and they are formed with PHF6 and PHF6^∗^ as a stable core. These interactions support previous observations of membrane-mediated toxicity and permeability, and they suggest tau has favorable interactions with phospholipid tails.

Finally, there is emerging evidence that tau forms condensates on anionic membrane surfaces. Cellular polyanions, such as RNA and microtubules, are known to promote tau phase separation, and early evidence suggests that tau forms a phase-separated gel formed on the surface of anionic membranes (Majewski et al., 2020). Phase-separated tau droplets have been observed surrounding the nuclear membrane, and these droplets may serve as nucleation sites for fibril growth and neurofibrillary tangles (Kang et al., 2020). The role of condensed tau is not yet clear, whether this will serve as a mechanism for reducing pathogenic aggregation, promoting aggregation, or simply concentrating tau at the membrane to promote its native functions. In comparison to the well-known factors controlling aqueous tau phase separation, the physical properties of membrane-nucleated tau condensates are yet to be elucidated.

In considering tau interactions with membranes, a few cautionary notes are advised to contextualize future research on the topic. The first is that it is still a matter of debate whether and how tau contributes to neurodegenerative diseases such as AD. The hypothesis that tau engages in prion-like propagation of toxic misfolded species is still under investigation. However, tau is clearly a useful hallmark of AD progression in the brain, and thus can serve as a powerful diagnostic tool. This is especially pertinent when considering detection of tau in cerebrospinal fluid and blood, where it will be important to consider how tau is accumulating extracellularly. Finally, there is the consideration of how tau interactions with membranes could potentially lead to cell toxicity. This could involve perturbation of membrane components leading to disruption of signaling pathways, disruption of membrane structure to cause leakiness, or induction of toxic aggregates of tau, especially β-sheet rich oligomers or fibrils. In exploring these possible toxic functions, it is important to consider the complex environment of the cell, including the many possible combinations of post-translational modifications to tau and the complicated composition of cellular membranes, including membrane-bound proteins.

In the future, it will be important to consider the role of the plasma membrane as an anionic surface in the context of the complex factors that regulate lipid domain organization. Several cellular factors are known to regulate membrane organization that could lead to regions of concentrated charge on the membrane surface beyond the 20% of anionic lipids expected in the cytosolic leaflet of the plasma membrane (Trimble and Grinstein, 2015). These factors include transmembrane junctions, the cytoskeleton, and lipid phase separation, which could each play a role in determining where tau is recruited to bind the membrane. Furthermore, it will be important to consider how the unique lipid and protein composition found in each intracellular membrane (i.e., nuclear, mitochondrial, ER, etc.) may impact tau interactions. Another area for further exploration is to update previous membrane-binding studies with the emerging knowledge that native tau is heavily phosphorylated. Future experiments should consider the use of phosphorylation mimicking forms as a better representation of native tau for studying interactions with membranes, especially as electrostatics play a critical role in tau-membrane interactions. Finally, the idea of membrane-induced tau condensation is only emerging and requires future exploration to elucidate the driving factors and cellular implications.

## Author Contributions

CV, CS, and BV reviewed the literature and wrote the review. CV, CS, and JM prepared the graphics. CS, BV, JM, JB, EM, EC, and CV contributed to the critical discussion and revision of the manuscript. All authors contributed to the article and approved the submitted version.

## Conflict of Interest

The authors declare that the research was conducted in the absence of any commercial or financial relationships that could be construed as a potential conflict of interest.

## Publisher’s Note

All claims expressed in this article are solely those of the authors and do not necessarily represent those of their affiliated organizations, or those of the publisher, the editors and the reviewers. Any product that may be evaluated in this article, or claim that may be made by its manufacturer, is not guaranteed or endorsed by the publisher.

## Abbreviations

AD: Alzheimer’s disease
PHF: paired helical filament
RD: repeat domain
PXXP: proline-X-X-proline
ANXA2: annexin A2
POPS: 1-palmitoyl-2-oleoyl-sn-glycero-3-phosphoserine
POPC: 1-palmitoyl-2-oleoyl-glycero-3-phosphocholine
DOPC: 1,2-dioleoyl-sn-glycero-3-phosphocholine
DOPG: 1,2-dioleoyl-sn-glycero-3-phospho-[1 ′ -rac-glycerol]
A β: amyloid-beta
LRP1: low-density lipoprotein receptor-related protein 1
DMPG: 1,2-dimyristoyl-sn-glycero-3-phospho-[1 ′ -rac-glycerol])
DMPS: 1,2-dimyristoyl-sn-glycero-3-phosphoserine
DMPC: 1,2-dimyristoyl-sn-glycero-3-phosphocholine
PI(4,5)P_2_: phosphatidylinositol 4,5-bisphosphate
LLPS: liquid–liquid phase separation.
